# Characteristics of Patients with Hospital Onset Clostridioides difficile Infections in a Safety Net Hospital

**DOI:** 10.1017/ash.2025.281

**Published:** 2025-09-24

**Authors:** Lujain Malkawi, Ume Abbas

**Affiliations:** 1 Infectious Diseases Department, University of Missouri-Kansas City; 2 University of Missouri- Kansas City

## Abstract

**Background:** Clostridioides difficile infections (CDI) are a leading cause of health-care associated morbidity and costs. University Health Truman Medical Center is a longstanding 238-bed safety net hospital in Kansas City, MO, where there was an increase in hospital-onset (HO) CDIs in 2024. To improve our infection prevention and control measures, we sought to study these HO CDI cases. **Methods:** Using a retrospective cohort study design and electronic health records, we retrieved data for inpatients who were identified as having HO CDI by our department of infection prevention and control in 2024. HO CDI was defined as a positive test for toxigenic Clostridioides difficile (C. difficile) polymerase chain reaction (PCR) performed on unformed stool collected on hospital day > 3 (with preagreed intuitional criteria in place). Data included demographic and epidemiological variables, comorbidities, onset of diarrhea and timing of stool collection, length of stay (LOS) and exposures (within prior 6 months) to hospitalization, surgery, and/or medications including laxatives, proton-pump inhibitors, immunosuppressants and antimicrobials. **Results:** In 2024 there were 20 HO CDI cases (versus 9 in 2023) with consequent increase in the CDI rate per 10,000 patient days and the standardized infection ratio. The characteristics of the CDI cases (percentage; mean ± standard deviation) were as follows. Most cases were females 60%. The mean age was 61 ± 18 years and BMI 28 ± 11 kg/m2. Recent hospitalization was common; 50% of cases had been hospitalized within 28 days and 70% within 6 months of their positive C difficile test. All cases had one or more comorbid conditions while one patient (5%) had past history of CDI. The median LOS was 18 days with frequent room changes and 35% of cases had an intensive care unit exposure. All had received systemic antibiotics either singly or in combination and the most commonly used agents included cephalosporins (90%) and penicillins with beta-lactamase inhibitor (35%). Laxative use was common (65%) as were history of surgery (55%) and intravenous contrast exposure (50%). Most cases (70%) were treated with oral vancomycin with three cases receiving a taper/prophylaxis, while five cases received fidaxomicin; there was one case of recurrence. **Conclusions:** Recent hospitalization and laxative use were high among HO CDI cases in a safety net hospital, raising concern for potential over-diagnosis. Switching to a two-step C difficile stool testing algorithm (PCR+ toxin enzyme immunoassay), though more costly, would be a useful mitigation strategy.

